# Growth performance and development of internal organ, and gastrointestinal tract of calf supplementation with calcium propionate at various stages of growth period

**DOI:** 10.1371/journal.pone.0179940

**Published:** 2017-07-10

**Authors:** Xinzhuang Zhang, Xin Wu, Wanbao Chen, Yawei Zhang, Yuming Jiang, Qingxiang Meng, Zhenming Zhou

**Affiliations:** State Key Laboratory of Animal Nutrition, College of Animal Science and Technology, China Agricultural University, Beijing, China; Gaziosmanpasa Universitesi, TURKEY

## Abstract

To investigate the effects of calcium propionate (**CaP**) supplementation on performance, the development of the internal organ, and gastrointestinal tract of calves at various stages of growth period, 54 male Jersey calves (age = 7 ± 1 d, body weight(BW) = 23.1 ± 1.2 kg) were randomly allocated to three treatment groups. While control calves were fed basis dietary with no additives (0CaP), other treatment calves were fed basis dietary supplementation with CaP at 50 (5CaP) or 100 (10CaP) g kg^−1^ dry matter. The experiment lasted 160 d and was divided into three feeding stages: Stage 1 (d 0 to 30), Stage 2 (d 31 to 90), and Stage 3 (d 91 to160). Six calves from each group were randomly selected and slaughtered on days 30, 90, and 160 when at the conclusion of each experimental feeding stage. The BW of calves increased with 10CaP after feeding 90 d, whereas it increased with 5CaP and 10CaP at feeding 120d and 160d compared to 0CaP. The 10CaP group improved average daily gain (ADG) of calves at stage 2, and d120-160 of stage 3 compared with the 0CaP group. The ADG of 5CaP was greater than the 0CaP group only at 120–160 d of stage 3 compared with the 0CaP group. The results of feed efficiency were in agreed with ADG as no dry matter intake difference at all stages of growth period. The 10CaP treatment exhibited the greatest spleen weight among the treatment at the end of the experiment; the liver weight of the 5CaP and 10CaP calves at feeding 90 d and of the 10CaP calves at feeding 160 d and were greater than those of the 0CaP animals. The CaP at the tested doses increased the rumen weight after feeding 90d of Jersey calves, and also improved the development of intestine. In conclusion, dietary supplementation with calcium propionate at the tested doses caused a beneficial effect in the growth performance and gastrointestinal tract traits of Jersey calves, thus to add 10% CaP before feeding 90 days was better and 5% CaP supplementation was expected at the period for feeding 90 to160 d.

## Introduction

Calcium Propionate (**CaP**) is approved as a mold inhibitor of food and feed by the World Health Organization and the United Nations Food and Agriculture Organization. Supporting this use, Sahib et al. (2010) found that the addition of CaP could suppress the germination, growth rate, and aflatoxin production of Aspergillus flavus [1]. Similarly, a 5g/1000 g dose of CaP reduced the total fungal count and aflatoxins B1, B2, G1 and G2 production in broiler starter feed [2]. In addition, along with its use in the feed industry, CaP is also used to preserve fresh fruits and vegetables to extend the shelf life and enhance nutritional value [3].

In highly productive livestock systems, alternative food sources should be selected in order to maximize energy consumption and growth performance [4]. Brown et al. (2005) found that increased energy intake can enhance the rate of body growth of heifer calves and potentially reduce rearing costs [5]. Fluharty et al. (1999) suggested that more energy and protein supply may contribute to the greater visceral organ mass in lambs[6]. Shen et al. (2004) reported that an energy-rich diet improved rumen papillae proliferation in young goats[7]. Therefore, dietary energy concentration is essential for growth performance and development of internal organ, and gastrointestinal tract of calf supplementation with calcium propionate at various stages of growth period.

Calcium Propionate is hydrolyzed to calcium ion and propionate acid in the rumen. Propionate is the primary precursor for glucose synthesis, and can supply up to 90% glucose for ruminants [8]. CaP is used to correct metabolic problems as a readily available energy source in dairy cattle [9]. Lee-Rangel et al. (2012) reported that CaP partially replaces the energy usually supplied by grain in diets for finishing lambs[10]. Zhang et al (2015) found that adding CaP had no effect on performance of finishing steers fed a high-concentrate diet [11]. Bunting et al. (1999) found that 6.4% CaP inclusion did not improve the ADG and intestinal development in 6 weeks of male Holstein calves[12], which the experimental period may have been too short to get significant results. The hypothesis of this study was that CaP could act as an energy source to improve performance and development of internal organs and the gastrointestinal tract of calves. Therefore, the objective of this study was to evaluate the effects of CaP supplement level on performance and development of internal organs and the gastrointestinal tract (**GIT**) of calves at various stages of growth period during 160 experimental days.

## Material and methods

The experiment was conducted at the experimental farm of the China Agricultural University and Beef Cattle Research Center in Daxing District, Beijing, China. Experimental procedures involving animals complied with the guidelines approved by the Animal Welfare Committee of China Agricultural University (Permit Number: DK15018) on experimental animals.

### Animals and experimental design

In total, 54 male Jersey calves were included in the experiment (average age and body weight upon arrival: 7 ± 1 days and 23.1 ± 1.2 kg respectively). All calves were purchased from the Sanyuan Xinlvzhou Dairy Farm to obtain a uniform group of calves of similar age. In the initial 3 days of life, all calves received colostrum only (2.0 L twice daily) via an esophageal feeding tube. Three days after weaning from colostrum, calves were readily drinking milk from buckets and showing no apparent health problems.Calves were selected and transported to the China Agricultural University Beef Cattle Practical Education Base, located in Daxing District, Beijing. Upon arrival, calves were divided into three groups of 18 animals based on initial body weight (BW) and were then housed in individual pens (1.5 × 2.5 m; bedded with wood shavings). Nose-to-nose contact between calves was minimized by pen arrangement. The calves were assigned randomly to one of three treatment groups: control calves were fed basis dietary with no additives (0CaP), other treatment calves were fed basis dietary supplementation with CaP at 50 (5CaP) or 100 (10CaP) g kg^−1^ dry matter. CaP supplementation was determined on a dry matter basis of milk, milk replacement and starter ration respectively. The CaP (purity = 99.26%) used in this experiment was purchased from Dongxin Chemical Plant (Tengzhouzhongxin Co.Ltd, Shandong, China).

### Diets and feeding management

The duration of the experiment was 160 d and was divided into three stages: Stage 1 (d 0 to 30), Stage 2 (d 31 to 90), and Stage 3 (d 91 to160). Calves were provided fresh water *ad libitum* throughout the experimental. In Stage 1, calves were fed milk at a daily rate of 10% of initial body weight. CaP was supplemented by mixing with milk. Weight was measured on weekly intervals to adjust the amount of milk required per animal. The milk was bucket-fed at 39°C in three equal feedings per day (0630, 1430, and 2030 h) during Stage 1. To guarantee the quality of the milk, milk was purchased daily from Sanyuan Lvmuyuan Dairy Farm, located less than 2km from experimental facility. During Stage 2, calves received a commercial (Nutrifeed, Inc., Friesland Campina, Netherlands) milk replacement (Table 1). The milk replacement was reconstituted with water at 40 ± 1°C. The replacement concentration in the liquid diet was 12.5%. In Stage 2, calves were weighed at monthly intervals, and the milk replacement powder was fed at 1.5% of initial BW, as recorded at the beginning of Stage 2. All calves were bucket-fed the milk replacement at 39°C three times per day (0700, 1400, and 2100 h) during Stage 2. After milk replacement feeding, a starter ration was made available to each calf. The detailed procedures of starter feeding are as follows: first, 100g starter ration was provided to each calf per day, if this amount was consumed then an additional 100g was provided. And then, the procedure steps into the next cycle. Daily feed intake for each calf was recorded. CaP was supplemented through mixing with both milk replacement powder and starter ration. Stage 3 lasted from 91 d to 160 d and all calves were weaned from milk replacement at the start of this stage. The starter ration feeding program of the schedule starter ration continued unchanged from Stage 2. In addition, to prevent rumen acidosis and facilitate animal welfare, all calves in Stage 3 were fed an equal amount of chopped alfalfa hay, provided after starter ration was supplied, the same amount chopped alfalfa hay, which was depended on completely consumed of all calves, was fed in stage 3 for preventing rumen acidosis and animal welfare. In Stage 3, CaP was only mixed with the starter ration. Calves were weighed before morning feeding at 120 d and 160 d respectively, and the feed intake was recorded daily. Calves were supplemented with 5% or 10% CaP was supplemented with 5% or 10% on a dry matter basis for the respective experimental groups during the different experimental stages.

**Table 1:** The ingredients of starter and the nutrient compositions of milk replacement, starter ration, and alfalfa hay.

Ingredients, % DM	Milk replacement	Starter ration	Alfalfa hay
Steam-flaked corn	-	50	-
Extruded full-fat soybeans	-	35	-
Wheat bran	-	10	-
Premix^1^	-	5	-
Nutrient composition, %DM			
Dry matter	96.5	89.9	92.2
NEm, Mcal/kg^2^	3.1	2.7	2.3
NEg, Mcal/kg^2^	2.2	1.9	1.6
Cure protein	22.4	19.7	16.5
Ether extract	18.6	9.1	2.4
Neutral detergent fiber	0.8	16.9	41.5
Acid Detergent Fiber	0.3	5.8	29.8
Ash	5.2	3.0	7.2
Calcium	0.8	0.9	1.4
Phosphorus	0.5	0.5	0.2

^1^The Premix was formulated to provide 100g calcium, 40g phosphorus, 150g salt, 18000IU VA, 4000IU VD_3_, 800IU VE, 4000mg Fe,300 mg Cu, 2100 mg Zn, 2000 mg Mn, 5000 mg S, 25 mg I, 10 mg Se and 20 mg Co per kilogram of DM.

^2^ calculated based on NRC (2000)

### Feed analysis

Samples of the milk replacement, starter feed, and alfalfa were collected every week and analyzed for DM, CP, crude fat, ash, calcium, and phosphorus by AOAC (1990) methods [13]. Feed samples were also analyzed for NDF and ADF content, as described previously [14]. The ingredients of the nutrient composition of milk replacement, the starter ration, and alfalfa hay are listed in Table 1, which NEm and NEg are calculated based on NRC 2000[15].

### Growth performance and sampling procedures

ADG of calves was calculated by dividing the difference between the final body weight, or an interval body weight, and the initial weight by the number of days (160 days, or an interval). Feed efficiency (Gain: Feed) was calculated as the ratio of individual average daily gain and dry matter intake.

There were three stages in this experiment, 0–30 days, 31–90 days and 91–120 days. At the end of each stage, six calves were randomly selected from each treatment. Feed was withheld for 12 hours before transportation to a commercial slaughterhouse 5km away. Transportation and slaughter procedures met current welfare guidelines. Stunning was performed with a captive bolt and was followed by jugular exsanguination. The gastrointestinal tract (GIT) and internal organs were immediately removed, the length of small intestine was measured and different compartments were separated by ligatures. Each compartment of GIT and internal organs were removed from the abdominal cavity, stripping all attachments, large blood vessels, lymph nodes and fat tissue. Two 2-cm sections were removed from the duodenum (within 10 cm of pylorus), jejunum (estimated midpoint of small intestine), and ileum (within 10 cm of cecum) were collected and fixed in 10% formalin solution in preparation for morphometric analysis of villus lengths and crypt depths immediately, according to He et al. (2015) [16]. The villus:crypt ratio was calculated as the villus length divided by crypt depth. The duodenum was only sampled from the 60 day period. The different stomach compartments: rumen, reticulum, omasum, and abomasum were weighed after removal of the ingesta and rinsing with cold water. Following dissection and thorough washing, wet weights were recorded for the internal organs, including heart, liver, spleen and kidney.

### Statistical analysis

The effects of dietary CaP supplementation on each stage on growth performance, the internal organs and the stomach development were evaluated using generalized linear model (GLM) procedures of SAS 9.0 (SAS Institute Inc., Cary, NC, USA). The mixed model procedure of SAS 9.0 (SAS Institute Inc., Cary, NC, USA) was used to analyze the effects of age and dietary CaP supplementation on small intestine development. The following mixed statistical model was used for analysis:

$$y_{\mathrm{ik}}=\mu +\alpha_{i}+\beta_{k}+{\left(\alpha \beta \right)}_{\mathrm{ik}}+e_{\mathrm{ijk}};$$

where y_ik_ is an observed value for length of small intestine, villus lengths and crypt depths of intestinal segment, and the villus:crypt ratio taken from the sample receiving treatment i at age k; μ is the overall mean; α_i_ is the fixed effect of treatment i; β_k_ is the fixed effect of age k; (α β)_ik_ is the interaction between treatment and age of calves; and e_ijk_ is the residual value.

The differences in treatment means were tested using Duncan’s multiple range tests. A level of *P* ≤ 0.05 was considered to be significant and 0.05 < *P* < 0.1 was considered as a tendency or trend.

## Results and discussion

### Growth performance

**Fig 1:**
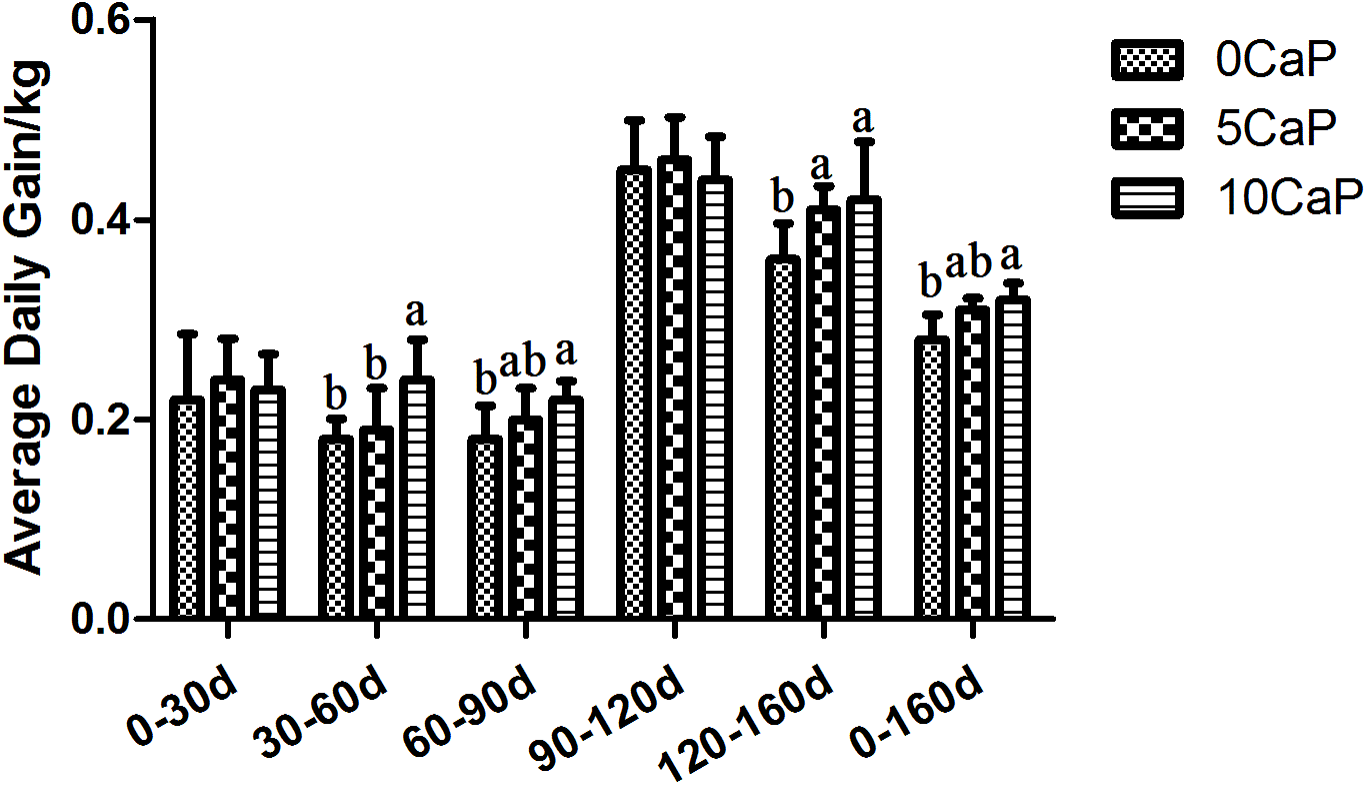
The ADG of calves supplemented with calcium propionate at various stages of growth period. Stage 1:0-30d; Stage 2:30-60d, 60-90d; Stage 3: 90-120d, 120-160d.

Fig 1 showed the ADG of calves supplemented with calcium propionate at various stages of growth period. There were no effects of CaP supplementation on ADG at Stage 1 (P = 0.649), in according to the results of Bunting et al. (2000), which found that adding 6.4% CaP also did not affect ADG over 6 weeks period of male Holstein calves. Adding 10% CaP improved the ADG of calves at stage 2, and 120–160 d of stage 3 compared to the 0CaP group (*P* <0.05). Whereas the 5CaP group only showed a positive effect between feeding 120–160 d (*P* <0.05) when compared to the 0CaP group. As shown in Table 2, an effect of calcium propionate supplementation on body weight was seen in the first at feeding 90 days, with the 10CaP group exhibiting higher weights than the 0CaP group. Over time the effect became more pronounced, and both the 5CaP and the 10CaP groups had higher body weights than the 0CaP group at feeding 120 days and 160 days (*P* <0.05). The 5CaP and 10CaP groups did not differ. The inconsistent results between ADG and BW was due to the accumulative effects of ADG of 10CaP group during feeding 30-90d contribute to greater BW until feeding 90d.

**Table 2:** The body weight, dry matter intake and feed efficiency of calves supplemented with calcium propionate at various stages of growth period.

Items	0CaP	5CaP	10CaP	SEM	*P* -value
Body weight(kg)					
0d	22.90	23.12	23.34	0.978	0.946
30d	29.59	30.55	30.11	0.962	0.750
60d	34.96	36.39	37.39	0.975	0.210
90d	40.42^b^	42.46^a^^b^	44.05^a^	0.794	0.013
120d	53.09^b^	56.16^a^	57.12^a^	0.927	0.018
160d	68.34^b^	72.73^a^	73.92^a^	1.183	0.007
Dry matter intake (kg/d)					
0-30d	0.31	0.32	0.32	0.006	0.442
31-60d	0.64	0.66	0.64	0.007	0.142
61-90d	0.89	0.91	0.88	0.012	0.223
91-120d	1.09	1.08	1.05	0.016	0.290
121-160d	1.28	1.28	1.27	0.008	0.275
0–160 d	0.87	0.88	0.86	0.010	0.274
Feed efficiency (kg gain: kg dry matter intake)						
0-30d	0.72	0.78	0.75	0.083	0.881
31-60d	0.28^b^	0.30^b^	0.38^a^	0.023	0.015
61-90d	0.21^b^	0.22^a^^b^	0.25^a^	0.012	0.042
91-120d	0.41	0.43	0.42	0.021	0.725
121-160d	0.29^b^	0.32^a^	0.33^a^	0.012	0.052
0-160d	0.33^b^	0.36^a^^b^	0.38^a^	0.009	0.036

^a-c^ Different superscripts within a row represent significant differences (*P* < 0.05); SEM: standard error of mean

Propionate is the primary precursor for glucose synthesis, and can be the precursor for up to supply 90% of rumen glucose production for ruminants. Propionate will provide additional, easily available energy for the calf, since it can be converted to glucose in the liver. Abeni et al (2000) reported that greater higher plasma glucose concentrations [17], means there is a amount of greater energy availability leading to and results in greater body increased weight gain. In the present study, it is therefore likely that the increasing glucose formation from the supplemented calcium propionate was the driving force behind the significant improvement growth performance seen in the calves of calves (P<0.05, Table 2) in the 5CaP and 10CaP groups.

The effects of CaP supplementation on DMI and feed efficiency in Jersey calves are presented in Table 2. There were no differences in DMI among treatments during the different stages (*P* > 0.05). In a previous study, Gilliland et al. (1962) found that the calves receiving the starter containing 10% VFA salts had decreased DMI, but no effect on BW [18]. Therefore, in the current experiment the lack of differences in DMI was expected to result in positive effects of CaP supplementation on growth performance of calves.

No effects of CaP supplementation on feed efficiency during Stage 1 (0–30d) (*P* = 0.880) and Stage 3 (90–120 d) (*P* = 0.725) among the treatments (*P* > 0.05) were observed. The feed efficiency of 10%CaP group was greater than the other groups during Stage 2 (30–60 d) (*P* < 0.05). During the Stage 2 (60–90 d), 10CaP group had the greater feed efficiency than 0CaP group, while 5CaP group was intermediate and did not differ from the other groups. The feed efficiency of 0CaP group at feeding 120–160 d was the least and differed from 5CaP and 10CaP group. The enhancement in ADG was consistent with the feed efficiency for the different stages because no differences in DMI were observed across the treatments.

CaP supplementation improved the growth performance but the effects were varied throughout the different experimental stages. Before feeding 30d, adding CaP had no effects; in stages 2, the positive effects on growth performance were enhanced with the level of CaP supplementation because higher levels of CaP supplementation could supply more propionate to synthesize. Positive effect of CaP supplementation on performance of calves in the latter part of the study was a result of increasing starter intake with calf age and thus increasing intake of CaP. Therefore, CaP supplementation was expected in starter rather than in milk in this study, which supplementation in starter results in propionate release in the rumen and its absorption into the blood whereas propionate supplementation in milk replacement results in its delivery to the abomasum and small intestine and most likely its metabolism by the epithelium of those regions of the gastrointestinal tract.

However, after feeding 90d in stages 3, adding either 5% or 10% calcium propionate improved the growth performance whereas and there was no difference between the 5CaP and 10CaP group. The lack of a dose related response may be explained by the findings of Zhang et al.(2015) who reported that no effects of CaP on ADG were expected when animals were fed high concentrate diets as they already supply sufficient propionate that further supplementation calcium propionate does not significantly increase propionate concentration in rumen[11].

### Internal organ development

Major functions of the internal organs are digestion and absorption of dietary nutrients, along with supplying a plethora of hormones and they form part of the immune response [19] (Huntington, 1990). Therefore, the weight of the internal organs reflects growth, health and general body condition. As shown in Table 3, CaP supplementation had no effect on the wet weight of the heart and kidneys during the experimental period (*P* >0.05). In contrast however, the 10CaP group exhibited the greatest spleen weight among the treatments at the end of Stage 3 (*P* < 0.05). The liver weight of the 5CaP and 10CaP group was greater than the 0CaP group (*P* < 0.05) at the end of Stage 2 (d 90). The 10CaP group maintained this difference until the end of the experiment. In contrast the liver weights of the 5CaP group were no longer greater by the end of the experiment. Similarly, Cao et al. (2014) demonstrated that the liver weight of geese fed with a high energy density diet was greater than the geese fed with a low energy density diet [20]. The increasing liver weight in the present study could metabolise more of the glucose precursors available due to the calcium propionate supplementation and therefore enhance gluconeogenesis.

**Table 3:** The internal organs development of calves supplemented with calcium propionate at various stages of growth period.

Items^1^	0CaP	5CaP	10CaP	SEM	*P* -value
Heart(kg)					
30d	0.22	0.24	0.24	0.010	0.328
90d	0.34	0.34	0.34	0.018	0.977
160d	0.42	0.43	0.46	0.012	0.062
Liver(kg)					
30d	0.73	0.72	0.82	0.031	0.121
90d	0.90^b^	1.05^a^	1.12^a^	0.039	0.003
160d	1.20^b^	1.30^a^^b^	1.39^a^	0.037	0.004
Spleen(kg)					
30d	0.09	0.10	0.10	0.008	0.615
90d	0.17	0.17	0.18	0.016	0.909
160d	0.24^b^	0.22^b^	0.27^a^	0.010	0.011
Kidney(kg)					
30d	0.14	0.13	0.14	0.007	0.395
90d	0.25	0.25	0.22	0.018	0.433
160d	0.31	0.31	0.28	0.014	0.292

^1^The weight of rumen, reticulum, omasum and abomasum were wet weight

^a-c^ Different superscripts within a row represent significant differences (*P* < 0.05); SEM: standard error of mean

### Gastrointestinal tract (GIT) development

Table 4 shows the effects of CaP supplementation on GIT development of different stages of growth. There was no difference for all measures at 30d among the treatment (*P* > 0.05). The reticulum weight was greater in the 5CaP and 10CaP groups than in the 0CaP group at the end of Stage 2 (90 d) (*P* = 0.016). The omasum weight of the 5CaP group was significantly heavier than the 0CaP group at 160 d (*P* = 0.047). The weight of the omasum was even greater for the 10CaP group significantly different to the other two groups at the same time point (*P* = 0.001).

**Table 4:** The stomach development of calves supplemented with calcium propionate at various stages of growth period.

Items^1^	0CaP	5CaP	10CaP	SEM	*P* -value
Rumen (kg)					
30d	0.19	0.20	0.23	0.022	0.286
90d	0.88^b^	1.12^a^	1.27^a^	0.062	0.006
160d	1.65^b^	1.95^a^	2.07^a^	0.094	0.010
Reticulum(kg)					
30d	0.05	0.06	0.06	0.005	0.217
90d	0.16^b^	0.23^a^	0.23^a^	0.014	0.016
160d	0.26	0.30	0.27	0.019	0.308
Omasum(kg)					
30d	0.07	0.06	0.07	0.005	0.496
90d	0.18	0.22	0.20	0.018	0.434
160d	0.37^b^	0.48^a^	0.44^a^^b^	0.030	0.047
Abomasum(kg)					
30d	0.19	0.20	0.22	0.014	0.546
90d	0.29	0.33	0.28	0.018	0.210
160d	0.32^b^	0.34^b^	0.44^a^	0.022	0.001

^1^ The weight of rumen, reticulum, omasum and abomasums were wet weight

^a-c^ Different superscripts within a row represent significant differences (*P* < 0.05); SEM: standard error of mean

Numerous studies have shown that short-chain fatty acids induce ruminal papillae development [21–23]. Liu et al. (2009) found that adding CaP promoted rumen fermentation of ruminally cannulated Chinese Simmental steers [24]. In addition, Shen et al. (2004) found that the level of diet energy was positively correlated with the development of rumen epithelium [7]. Therefore, supplementation with CaP would likely lead to increased rates of ruminal papillae and epithelial development, providing the mechanism behind the significantly increased the rumen weight after 90d of calves (*P* = 0.006, 0.010) supplemented with calcium propionate. Similarly, Xie et al. (2013) also revealed that calves with a greater concentration of propionate through fed a mixture of steam-flaked corn and extruded soybeans, showed a better rumen development compared the calves fed only milk replacer [25].

Propionates improvement of rumen development and could be interpreted through two aspects. Propionate could stimulate the release of insulin [26], which could stimulate the mitosis of rumen epithelial [27]. Alternatively, propionate stimulates rumen development directly as a signaling molecule because propionate was the most sensitive specificity ligand for G protein-coupled receptors 41 and 43 [28] and G protein-coupled receptor 43 was exited in rumen epithelium reported by Wang et al. (2012) [29].

### Intestinal development and morphological measurements

As shown in Table 5, there were no differences on intestinal length among the treatments (*P* = 0.153). The intestinal length at the end of Stage 3 (160 d) of calves was greater than at the end of Stage 1 (30d) and Stage 2 (90d) (*P* < 0.001). The 5CaP and 10CaP groups had greater villus height (*P* = 0.007) and crypt depths (*P* = 0.0002) in duodenum compared to the 0CaP group, whereas there was no difference across the treatments for the villus:crypt (*P* = 0.286). The crypt depths in the duodenum of calves at the end of Stage 3 were less than at the end of Stage 2 (*P* = 0.0005). The villus height (*P* = 0.004), crypt depths (*P* = 0.006) and the villus:crypt ratio in the jejunum (*P* = 0.008) increased with the level of CaP supplementation, which adding 10% CaP was greater compared to the 0CaP group(*P* <0.05). No effects of calves age was found on villus height, crypt depths and the villus:crypt ratio in the jejunum (*P* > 0.05). Calcium propionate supplementation increased the villus height in the ileum compared to the 0CaP group (*P* < 0.001). Moreover, 10CaP groups had greater crypt depths in ileum than 5CaP and 0CaP groups (*P* <0.001). Both villus height (*P* <0.001) and crypt depths (*P* = 0.0007) in ileum at the end of Stage 2 (90 d) and Stage 3 (160d) were greater than 30 d calves. However, there were no differences on the villus:crypt ratio between different ages (*P* = 0.697).

**Table 5:** Effects of CaP supplementation and ages on intestinal development and morphological measurements of calve.

Items	Treatments			SEM	Age			SEM	*P* ^1^-value		
	0CaP	5CaP	10CaP		30d	90d	160d		*P*_T_	*P*_A_	*P*_TXA_
Intestinal length (m)	21.71	22.95	22.16	0.442	19.58^b^	20.86^a^	26.38^a^	0.425	0.153	<0.001	0.066
Duodenum											
Villus height, μm	171.74^b^	187.38^a^	192.79^a^	4.040	-^2^	190.36	195.44	3.156	0.007	0.950	0.127
Crypt depths, μm	94.20^b^	99.97^a^	110.17^a^	2.208	-	107.37^a^	95.52^b^	1.724	<0.001	<0.001	0.001
Villus:Crypt	1.83	1.88	1.78	0.044	-	1.79	1.86	0.040	0.286	0.190	0.027
Jejunum											
Villus height, μm	164.67^b^	185.21^a^^b^	208.85^a^	3.190	190.61	200.25	187.38	11.945	0.004	0.759	0.114
Crypt depths, μm	90.88^b^	99.19^a^^b^	107.17^a^	3.271	93.04	103.49	100.70	3.263	0.006	0.087	0.405
Villus:Crypt	1.81	1.87^a^^b^	1.95^a^	0.033	1.84	1.94	1.86	0.028	0.008	0.056	0.001
Ileum											
Villus height, μm	177.58^b^	194.51^a^	204.65^a^	3.722	169.01^b^	202.73^a^	204.99	3.568	<0.001	<0.001	0.067
Crypt depths, μm	95.89^b^	100.70^b^	107.92^a^	2.518	91.97^b^	110.17^a^	110.17	2.414	<0.001	0.001	0.274
Villus:Crypt	1.85	1.93	1.83	1.864	1.86	1.87	1.87	0.032	0.103	0.697	0.931

^1^*P*_T_: *P* value of treatments; *P*_A_: *P* value of Jersey calves ages; *P*_TxA_: *P* value of the interaction between treatments and Jersey calves ages

^2^ The missing data of duodenum at 30d

^a-c^ Different superscripts within a row represent significant differences (P < 0.05); SEM: standard error of mean

The results of the present study suggested that adding CaP improved intestine development. The underlying mechanism of the positive effect on the intestine is likely to be similar to that of the rumen due to the large amount of propionate, 93 to 97% which exit the rumen [30]. In addition, the increase in available energy supplied by the CaP could contribute to the improvement of intestinal development. However, inconsistent with our study, Bunting et al. (1999) found that the 6.4% CaP inclusion did not improve the intestinal development [12]. The length of experimental period (160 d vs 42 d) is likely to be responsible to the different results as 42 days may not have been sufficient for measurable intestinal development effects.

## Conclusions

In summary, dietary supplementation with calcium propionate at the tested doses caused a beneficial effect in the growth performance and gastrointestinal tract traits of Jersey calves, thus to add 10% calcium propionate before feeding 90 days was better and 5% calcium propionate supplementation was expected at the period for feeding 90 to160 d.

## Supporting information

S1 File The data of development of internal organ and stomach. (XLS)

S2 File The data of intestinal development and morphological measurements. (XLSX)
